# Lyophilized Platelet–rich plasma for the management of thin endometrium and facilitation of *in-vitro* fertilization

**DOI:** 10.5935/1518-0557.20220021

**Published:** 2023

**Authors:** Buvaneswari Gangaraju, Pradeep Mahajan, Swetha Subramanian, Ajit Kulkarni, Sanskruti Mahajan

**Affiliations:** 1 Certified Console Surgeon, GBR Fertility Center and Hospitals, Mogappair, Chennai, TamilNadu, India; 2 Chairman and Managing Director, StemRx Bioscience Solutions Pvt. Ltd., Mumbai, Maharashtra, India; 3 Clinical Research Associate, StemRx Bioscience Solutions Pvt. Ltd., Navi Mumbai, Maharashtra, India; 4 Lab in-charge, StemRx Bioscience Solutions Pvt. Ltd., Mumbai, Maharashtra, India; 5 Research Tech, Dept. of Surgery, Indianapolis University School of Medicine, USA

**Keywords:** platelet-rich plasma, embryo transfer, reproductive, endometrium, thin

## Abstract

**Objective:**

Adequate endometrial thickness has been considered an important parameter for hormonal response and blastocyst implantation in assisted reproduction therapies. While there is no consensus on the exact thickness of the endometrium considered ‘adequate,’ a thin endometrium (<7mm) has been associated with compromised outcomes in assisted reproduction therapies. Platelet-rich plasma (PRP), which is a concentrate obtained from peripheral blood, is a rich source of growth factors that play important roles in various cellular processes. The objective is to utilize lyophilized PRP (LPRP) to increase the thickness of the endometrium and enhance the outcomes of embryo transfer in women with poor response to previous *in-vitro* fertilization procedures

**Methods:**

This study enrolled nine women between 23 and 42 years of age, with a thin endometrium, who had undergone multiple previous unsuccessful assisted reproduction procedures. All patients underwent intrauterine infusion of LPRP, followed by frozen-thawed embryo transfer after 2-3 days.

**Results:**

Endometrial thickness was assessed by ultrasound 2 weeks after LPRP infusion, which showed improved thickness in all patients (range, 0.7-2.2mm). Clinical pregnancy occurred in all patients and eight out of nine patients are currently between 9 weeks and 27 weeks of gestation. Twin fetal heartbeats were not detected at the eighth week in one patient.

**Conclusion:**

Infusion of LPRP was found to be beneficial to increase endometrium thickness in all patients. This regenerative technique could be considered to enhance the outcomes of assisted reproduction techniques in a minimally-invasive manner, without any side effects.

## INTRODUCTION

The field of regenerative medicine and cell-based therapy has explored the role of blood-derived biomolecules, including platelets, to simulate physiological coagulation events as well as to achieve wound healing and repair hard and soft tissues (Burnouf *et al*., 2013). Platelet concentrates are used in reproductive medicine, wherein translational research has paved the way for combined molecular/cellular and assistive reproductive techniques to overcome infertility.

Platelets or thrombocytes are small, non-nucleated cells produced by controlled fragmentation of multinucleated megakaryocytes residing in the bone marrow (Weiss, 1975). The role of platelets in activation of the coagulation cascade and ultimately blood clotting is well known. However, platelets also play a role in immune responses, angiogenesis, and wound and tissue healing. Furthermore, studies have reported that platelets act as growth factor reservoirs (present in their α-granules) that are released in response to injury (Ross *et al*., 1974; Savage & Cohen, 1972; Childs *et al*., 1982). The growth factors and cytokines present in platelets include platelet-derived growth factor (PDGF), epidermal growth factor (EGF), transforming growth factor (TGF), vascular endothelial growth factor (VEGF), platelet factor interleukin (IL), platelet-derived angiogenesis factor, IL-8, insulin-like growth factor, connective tissue growth factor (CTGF), and fibronectin.

Among the various causes of female infertility and conception failure, a thin endometrium has been considered an ongoing challenge (Lebovitz & Orvieto, 2014). Endometrial thickness has been directly correlated to increasing circulating estrogen levels and may be considered a predictor of success in assisted reproductive techniques (Hershko-Klement & Tepper, 2016). While there remains controversy regarding the exact definition and significance of a thin endometrium (commonly <7mm on the day of ovulation or human chorionic gonadotropin administration in cases of assisted reproduction), its role in blastocyst implantation has led to the need for therapies to improve its thickness for successful conception and pregnancy maintenance (Liu *et al*., 2019).

Platelet-rich plasma (PRP), which is a concentrate obtained from blood, contains a high number of platelets in a small volume of plasma, and has been utilized in the management of thin endometrium. Intrauterine infusion of PRP was reported to induce endometrial growth and maintain normal pregnancy in women with thin endometrium and poor response to conventional therapy during frozen-thawed embryo transfer (FET) cycles (Chang *et al.,* 2015).

While the method to obtain PRP is not technique-sensitive, there is no general consensus on the optimal protocol, and the preparation characteristics depend on the commercial system used (Castillo *et al*., 2011). Moreover, immediate availability of the systems in a clinical setting, requirement of additional manpower, and possible contamination during handling are the challenges in the use of fresh PRP. Maintenance of the integrity and function of platelets is paramount during PRP preparation. Therefore, the concept of lyophilization or freeze-drying PRP was introduced in order to standardize the preparatory process, while ensuring quality control; thereby maintaining the biological activity of the platelets and its constituent growth factors. The process also facilitated the logistics and storage-related aspects of the platelet products, thereby widening its scope of application.

In this report, we present the management of thin endometrium with lyophilized PRP (LPRP) infusion, prior to embryo transfer, in women with poor response to previous *in-vitro* fertilization (IVF) procedures.

## MATERIALS AND METHODS

### Patient characteristics

Nine women aged between 23 and 42 years, who visited GBR fertility clinic, Chennai, India with the chief complaint of inability to conceive or maintain pregnancy were included in this study. Inclusion of patients for this study was based on previous IVF failure and the presence of a thin endometrium, as seen on ultrasound. Three women had a previous history of abortion and three had polycystic ovary disease. Table 1 presents an overview of the included patients.

**Table 1:** Patient characteristics.

**Patient** **number**	**Age (years)**	**Abortion** **history**	**Previous IVF failure**	**History of** **PCO**	**Hb level** **(g/dL)**
1	44	1	1	No	12.8
2	33	2	1	Yes	12.1
3	26	1	2	Yes	11.9
4	33	0	2	No	13
5	23	0	0	Yes	13.1
6	39	0	0	No	12.1
7	30	0	1	No	13.8
8	34	0	1	No	11.5
9	41	0	3	Yes	12.3

IVF=*in-vitro* fertilization; PCO=polycystic ovary; Hb=hemoglobin

### LPRP

LPRP was manufactured at StemRx Bioscience Solutions Pvt. Ltd., Mumbai, India. Platelet bags were obtained from the blood bank and were subjected to three freeze-thaw cycles. Following this, the content was centrifuged and the supernatant was filtered under sterile conditions, which was then stored at -80°C for 4 hours. The lyophilizer was then set to reach a temperature between -45°C and -50°C, and the vials were placed inside the chamber. The vacuumeter was then started and the vials were lyophilized for 20-24 hours, until there was no moisture left in the product. Sealing and packaging was done under sterile conditions in a biosafety cabinet, following which the product was ready for use.

### Clinical procedure

Written informed consent was obtained from all patients prior to LPRP infusion. Eight patients underwent a single intrauterine infusion of LPRP (0.8 mL), while one underwent the procedure twice, considering the added requirement. A measured dose of 0.8 mL of water for injection was taken in a 1 mL syringe and was slowly diffused into the LPRP vial. The contents were gently mixed until the LPRP was completely diluted. This diluted solution was transfused into the endometrium within 15 minutes of preparation.

We placed the patients in the lithotomy position and used the CUSCOS speculum to visualize the cervix. The vagina was cleaned using povidone iodine and normal saline solution. An embryo transfer (ET) catheter was attached to a 1 mL syringe and filled with the prepared LPRP solution, which was then advanced through the cervix and internal os under ultrasound guidance. When the tip of the ET catheter was 5 cm below the fundus, the piston was gradually advanced to allow a steady flow of the LPRP solution into the uterine cavity. The catheter was gradually withdrawn till the tip reached the mid cavity of the uterus, and the piston was re-advanced. Continuous pressure was maintained to avoid backflow. The patients were instructed to maintain the head low position for 30 to 45 minutes, following which they were discharged.

All patients were prescribed incremental doses of estrogen (up to 16mg), progesterone vaginal gel 8%, dydrogesterone 10mg twice/thrice daily, a single dose of immunoglobulin, and multivitamins. Standard luteal support was provided to all patients.

**Figure 1:**
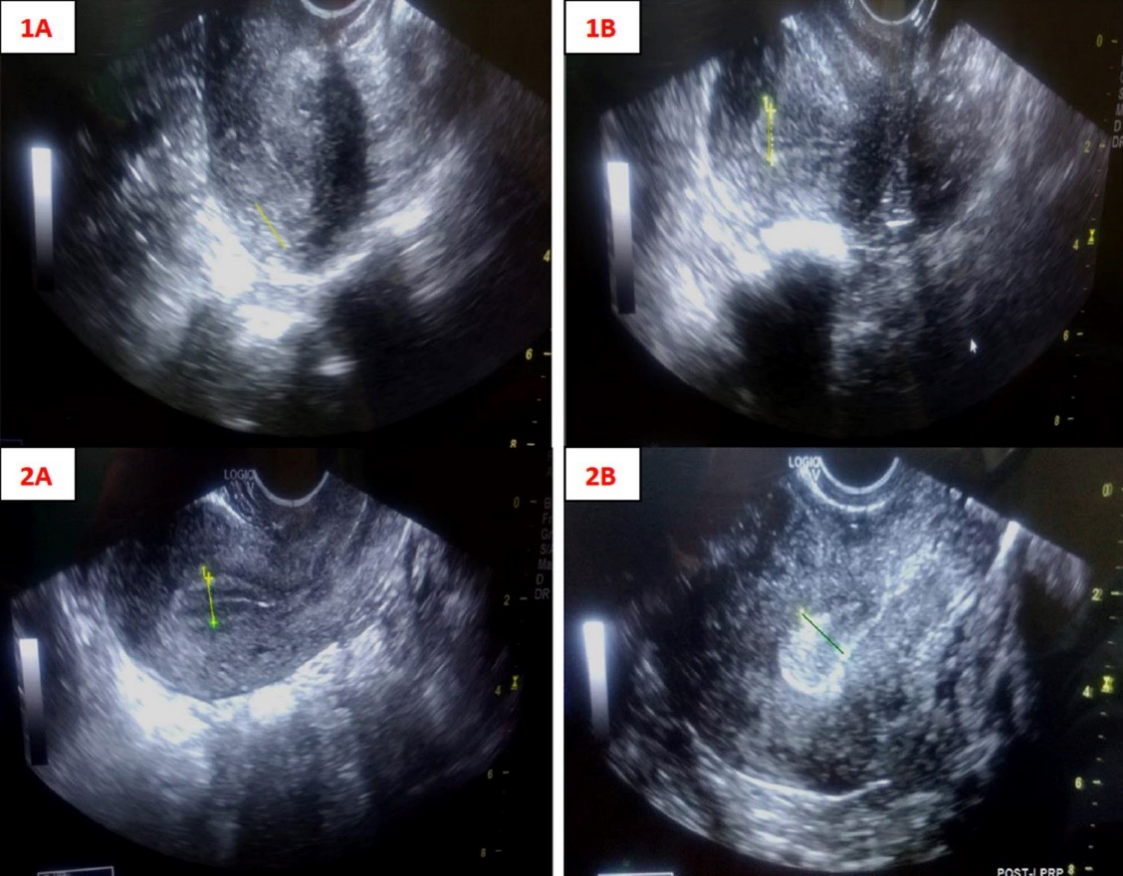
Pre- and post-LPRP treatment endometrial thickness of two representative patients. 1A: Pre-treatment endometrial thickness 5.9 mm (faint yellow line); 1B: Post-LPRP infusion endometrial thickness 8.1 mm (yellow line); 2A: Pre-treatment endometrial thickness 8.7 mm (yellow line); 2B: Post-LPRP infusion endometrial thickness 11.7 mm (green line).

We assessed endometrial thickness using ultrasound 2 weeks after the LPRP infusion. Figure 1 shows the pre- and post-LPRP treatment endometrial thickness of two representative patients. FET was done in each patient 2-3 days after the infusion.

## RESULTS

Table 2 shows the changes in endometrial thickness before and after LPRP infusion. None of the patients experienced any adverse effects following LPRP infusion. The progesterone levels in the patients before FET ranged from 0.17-1.46 ng/mL, which was within the favorable range to ensure increased pregnancy rates in the IVF cycle. Clinical pregnancy occurred in all patients and eight out of nine patients are currently between 9 weeks and 27 weeks of gestation. In one patient (case 6), the twin fetal heartbeats were not detected at the eighth week. In another patient (case 5), the heartbeat was absent in one fetus at the tenth week; however, the other fetus appeared normal. Table 3 shows the progesterone levels and gestational outcomes of the patients.

**Table 2:** Endometrial thickness before and after LPRP administration.

**Patient number**	**ET before LPRP (mm)**	**ET after LPRP (mm)**
1	6.2	7.0
2	8.5	9.5
3	8.0	8.7
4	7.3	9.5
5	6.2	7.8
6	7.0	9.0
7	5.0	6.4
8	5.9	8.1
9	8.7	11.7

ET=endometrial thickness; LPRP=lyophilized platelet-rich plasma

**Table 3:** P4 levels and gestational outcomes.

**Patient** **number**	**P4 level** **before FET**	**Clinical** **Pregnancy**	**Single/Twins**	**Weeks**	**Remarks**
1		Positive	Single	20	-
2	0.73	Positive	Twins	27	-
3	0.17	Positive	Single	16	-
4	0.96	Positive	Twins	16	-
5	0.42	Positive	Twins	13	One fetal heartbeat absent in the eighth week
6	0.24	Positive	Twins	8	Twin fetal heartbeats absent
7	1.46	Positive	Single	9	-
8	0.88	Positive	Single	14	-
9	1.01	Positive	Single	14	-

P4=progesterone; FET=frozen-thawed embryo transfer

## DISCUSSION

The rate of blastocyst implantation has been considered to increase in the presence of adequate endometrial thickness. The possible causes of a thin endometrium are low estrogen levels, fibroids, Asherman’s syndrome, poor blood flow, pelvic inflammatory disease, and chronic infections, among others. Liu *et al*. (2018) studied 18,900 FETs and found that the transfers occurred in 14.1% cases with endometrial thickness <8mm and in 3.1% cases with thickness <7mm. They concluded that although pregnancy and live birth rates decreased in cases of thin endometria (with each mm affecting the outcomes), reasonable outcomes were obtained even with lower endometrial thickness. Nonetheless, hormone replacement therapy and FET appear to demonstrate enhanced outcomes in cases with better endometrial receptivity. Thus, attempts have been made to promote endometrial growth, especially in cases of multiple IVF failures.

Several treatments, including extended estrogen therapy, adjuvant therapy with low dose aspirin, vaginal sildenafil, among others, have been considered for the treatment of thin endometrium with variable results (Barad *et al*., 2014; Groenewoud *et al*., 2013). Intrauterine infusion of granulocyte-colony stimulating factor (G-CSF), which stimulates neutrophilic granulocyte differentiation and proliferation, has been reported to promote endometrial proliferation and growth and has currently gained popularity (Gleicher *et al.,* 2011). However, considering the presence of growth factors and cytokines in PRP, combined with the ease of access and low immunological reactions compared to G-CSF, the former has been favored to promote endometrial growth.

Colombo *et al*. (2017) conducted a study on eight women with three cancelled cryo-transfers due to poor endometrial growth (<6mm). Following infusion of PRP, they reported an improvement in endometrial thickness to an average of 6.9 mm in seven patients, and a positive β-HCG test in six patients. They concluded that PRP administration could improve the multiple implantation failures caused by inefficient expression of adhesion molecules. Similarly, Zadehmodarres *et al*. (2017) reported an increase in endometrial thickness to >7mm at 48 hours after the first application in ten women with a history of cancelled embryo transfers. They concluded that PRP was effective in cases of refractory and thin endometrium and facilitated embryo transfer.

The clinical efficacy of platelet concentrates depends on the number of platelets and the concentration of their growth factors, which act as transmitters in tissue healing and morphogenesis (Lubkowska *et al*., 2012). A study demonstrated that the amount of intact platelets was higher in lyophilized as compared to fresh PRP (54% *vs*. < 20%, respectively) (da Silva *et al*., 2018). In addition, lyophilization prolongs the shelf life and facilitates storage, thereby making it more beneficial in clinical settings where the facility to obtain fresh PRP may not be available. Shiga *et al*. (2017) reported on the stability of freeze-dried PRP for up to 8 weeks at room temperature.

In the present study, positive pregnancy outcomes were observed in eight out of nine cases treated with LPRP prior to FET. A definitive increase in endometrial thickness of approximately 0.7-2.2 mm was seen in all patients. We know that the endometrium is composed of a cell-rich connective tissue stroma containing a rich supply of blood vessels. Among the different growth factors in LPRP, PDGF, VEGF, TGF, CTGF, and EGF, through their autocrine and paracrine properties, predominantly promote cellular activity (migration, proliferation, and differentiation) and extracellular matrix formation, as well as enhance angiogenesis (Samy *et al*., 2020). These properties contribute to an increase in endometrial thickness, as evidenced in this study. Thus, LPRP infusion may be considered a safe and effective therapeutic option to improve the outcomes of FET in thin endometrium patients.

Despite the predominantly positive outcomes, the present study has certain limitations. First, the small sample size and the single-center design, which may not adequately represent the population as a whole undergoing IVF procedures. Further multicenter studies with large sample sizes are required to generalize the results of our study. Second, the majority of our patients are in the first and second trimesters; therefore, the pregnancy progression, delivery-related, and postpartum outcomes could not be assessed in this study. A follow-up study will be planned, to include the aforementioned outcomes to provide more complete information on the effectiveness of LPRP.

## CONCLUSION

Intrauterine LPRP infusion promoted endometrial growth and enabled FET, which could not be previously performed successfully due to thin endometria. The procedure was well-tolerated and clinical pregnancy was established in all patients. Thus, a regenerative medicine-based approach may be considered beneficial to enhance FET outcomes.
